# Routine innominate artery cannulation for elective ascending aortic surgery. A single-centre experience.

**DOI:** 10.1186/1749-8090-10-S1-A265

**Published:** 2015-12-16

**Authors:** Charilaos-Panagiotis Koutsogiannidis, Fotini Ampatzidou, Athanasios Madesis, Theodoros Karaiskos, Olga Ananiadou, Konstantinos Diplaris, Athanasios Malamas, George Drossos

**Affiliations:** 1 Cardiothoracic Surgery Department, General Hospital "G. Papanikolaou", Thessaloniki, Greece

## Background/Introduction

Innominate artery cannulation is indicated in operations for acute and chronic aortic disease and also in case of porcelain aorta or reoperations. We routinely used innominate artery cannulation in 81 elective operations which included ascending aorta replacement.

## Aims/Objectives

Aim of our study is to determine whether routine innominate artery cannulation is safe and effective for elective surgical procedures including ascending aorta replacement.

## Method

We excluded from the study all patients who underwent emergent or urgent operation for acute aortic syndrome, any aortic surgery distal to innominate artery and patients who had other than innominate artery cannulation (aortic, subclavian/axillary, femoral). The final cohort was consisted of 81 patients who underwent elective ascending aorta replacement alone or with concomitant procedures such as aortic root replacement, coronary or/and valve surgical interventions, which were performed only with innominate artery cannulation. Open distal anastomosis after clamping of the aortic arch branches and selective antegrade cerebral perfusion with a flow rate of 10 ml/kg/min, was performed in all patients. 43 peri-operative variables have been investigated.

## Results

The operations that have been performed are categorized into ascending aorta replacement with interposition graft (n = 27, 33.3%), Bentall procedure (n = 20, 24.7%), Bentall plus coronary artery bypass surgery (n = 8, 9.9%), ascending aorta replacement with interposition graft plus aortic valve replacement (n = 12, 14.8%), ascending aorta replacement with interposition graft plus coronary artery bypass surgery (n = 9, 11.1%) and ascending aorta replacement with interposition graft plus aortic valve replacement plus coronary artery bypass surgery (n = 5, 6.2%). In hospital mortality rate was 1.2% (n = 1). Three patients (3.7%) had postoperative stroke, 2 had transient ischemic attack (2.5%) and 7 developed cognitive dysfunction (8.6%). Average antegrade cerebral perfusion period was 20 minutes at a temperature of 29.4 oC. In three cases bilateral (left common artery) antegrade cerebral perfusion was also used.

## Discussion/Conclusion

Innominate artery cannulation performed for elective surgical procedures including ascending aorta replacement is safe and effective. It poses low mortality rate and a low risk of neurological events and cognitive dysfunction.

